# Treatment benefit among migraine patients taking fremanezumab: results from a post hoc responder analysis of two placebo-controlled trials

**DOI:** 10.1186/s10194-020-01212-4

**Published:** 2021-01-07

**Authors:** Stephen D. Silberstein, Joshua M. Cohen, Ronghua Yang, Sanjay K. Gandhi, Evelyn Du, Adelene E. Jann, Michael J. Marmura

**Affiliations:** 1 Jefferson Headache Center, Thomas Jefferson University, Philadelphia, PA USA; 2 Teva Branded Pharmaceutical Products R&D, Inc., West Chester, PA USA; 3 Department of Neurology, NYU Langone Health, New York, NY USA

**Keywords:** Fremanezumab, Monoclonal CGRP antibody, Preventive migraine treatment, Responder analysis

## Abstract

**Background:**

Monoclonal antibodies targeting the calcitonin gene-related peptide (CGRP) pathway, including the fully humanized monoclonal antibody (IgG2Δa) fremanezumab, have demonstrated safety and efficacy for migraine prevention. Clinical trials include responders and nonresponders; efficacy outcomes describe mean values across both groups and thus provide little insight into the clinical benefit in responders. Clinicians and their patients want to understand the extent of clinical improvement in patients who respond. This post hoc analysis of fremanezumab treatment attempts to answer this question: what is the benefit in subjects who responded to treatment during the two, phase 3 HALO clinical trials?

**Methods:**

We included subjects with episodic migraine (EM) or chronic migraine (CM) who received fremanezumab quarterly (675 mg/placebo/placebo) or monthly (EM: 225 mg/225 mg/225 mg; CM: 675 mg/225 mg/225 mg) during the 12-week randomized, double-blind, placebo-controlled HALO EM and HALO CM clinical trials. EM and CM responders were defined as participants with a reduction of ≥ 2 or ≥ 4 monthly migraine days, respectively. Treatment benefits evaluated included reductions in monthly migraine days, acute headache medication use, and headache-related disability, and changes in health-related quality of life (HRQoL).

**Results:**

Overall, 857 participants from the HALO trials were identified as responders (EM: 429 [73.8%]; CM: 428 [56.7%]). Reductions in the monthly average number of migraine days were greater among EM (quarterly: 5.4 days; monthly: 5.5 days) and CM (quarterly: 8.7 days; monthly: 9.1 days) responders compared with the overall population. The proportion of participants achieving ≥ 50% reduction in the average monthly number of migraine days was also greater in responders (EM: quarterly, 59.8%; monthly, 63.7%; CM: quarterly, 52.8%; monthly, 59.0%) than in the overall population. Greater reductions in the average number of days of acute headache medication use, greater reductions in headache-related disability scores, and larger improvements in HRQoL were observed among EM and CM responders compared with the overall populations.

**Conclusions:**

Fremanezumab responders achieved clinically meaningful improvements in all outcomes. The magnitude of improvements with fremanezumab across efficacy outcomes was far greater in responders than in the overall trial population, providing insight into expected treatment benefits in participants who respond to fremanezumab in clinical practice.

**Trial registration:**

ClinicalTrials.gov identifiers: NCT02629861 (HALO EM) and NCT02621931 (HALO CM).

## Background

Calcitonin gene-related peptide (CGRP) plays a major role in migraine pathophysiology [1]. Monoclonal antibodies targeting the CGRP ligand or its receptor are effective and safe in the prevention of migraine [1, 2]. However, the full extent of their benefits has not been fully characterized.

Treatment benefit, as assessed by the treatment response rate, is typically measured as reduction from baseline in a headache outcome (e.g., reduction of the number of migraine days or of moderate to severe headache days) [3]. Since treatment effects vary depending on responder status, this may inadequately capture their benefits in responders. Although optimal responder rates for migraine preventive therapies have not been established, a response rate of ≥ 50% reduction from baseline is often used as a clinically significant threshold for participants with episodic migraine (EM); other response rates (e.g., ≥ 75% and 100%) may also be used to quantify even greater treatment benefits [3–5]. Due to the severity of symptoms and disease for participants with chronic migraine (CM), a lower response rate threshold of ≥ 30% may be clinically meaningful [4].

Fremanezumab is a fully humanized monoclonal antibody (IgG2∆a) that selectively targets CGRP; it is approved in the United States, the European Union, and many other countries for the preventive treatment of migraine in adults [2, 6, 7]. In previous placebo-controlled clinical trials, fremanezumab demonstrated efficacy as a preventive treatment for both EM and CM, with a favorable safety and tolerability profile [8, 9]. Moreover, results from the two 12-week phase 3 HALO EM and HALO CM clinical trials demonstrate significant improvements in the number of monthly average migraine days (MAMD) among participants receiving fremanezumab quarterly or monthly compared with those receiving placebo [10, 11].

Treatment effects, by definition, are worse in those who do not respond to treatment compared to those who do. Healthcare professionals and people with migraine may have particular interest in the extent of improvement among responders, rather than those who do not respond, as responders would continue on therapy and accumulate the intended preventive benefits. However, clinical trial results published to date do not provide data on improvements achieved among responders. As such, the goal of this post hoc analysis was to evaluate the treatment benefit of fremanezumab among people with migraine who responded to treatment during the HALO EM and HALO CM trials [10, 11]. Treatment benefit outcome measures included migraine days, headache days of at least moderate severity, acute headache medication use, headache-related disability, and overall health-related quality of life (HRQoL).

## Methods

### Study design

The HALO EM (NCT02629861) and HALO CM (NCT02621931) trials were both 12-week, randomized, double-blind, placebo-controlled, parallel-group studies. The designs and primary results for these 2 trials have been described previously [10, 11]. Study inclusion criteria were a history of migraine (as defined by the International Classification of Headache Disorders, 3rd edition [beta version; ICHD-3 beta]) for ≥ 12 months as well as fulfillment of the criteria for EM or CM during the 28-day pretreatment period. EM was defined as a headache occurring on 6 to 14 days, with ≥ 4 fulfilling ICHD-3 beta criteria for migraine with or without aura, probable migraine, or use of triptans or ergot derivatives [10]. CM was defined as headache of any duration or severity on ≥ 15 days and headache meeting ICHD-3 beta criteria for migraine on ≥ 8 days [11]. A subset of participants were permitted to use 1 concomitant preventive migraine medication provided that the dosing was stable for ≥ 2 months prior to the beginning of the pretreatment period and there was no change in dose during the study. Participants were permitted to use acute headache medications.

Participants who met eligibility criteria were randomized 1:1:1 to receive subcutaneous injections of fremanezumab quarterly, fremanezumab monthly, or placebo. Placebo dosing consisted of three 1.5 mL injections at baseline, one 1.5 mL injection at Week 4, and one 1.5 mL injection at Week 8. Quarterly dosing consisted of fremanezumab 675 mg (three 225 mg/1.5 mL injections) at baseline and placebo (single 1.5 mL injections) at Weeks 4 and 8. For participants with EM, monthly dosing consisted of fremanezumab 225 mg/1.5 mL (with two 1.5-mL placebo injections to maintain the blind) at baseline and single fremanezumab 225 mg/1.5 mL injections at Weeks 4 and 8. For participants with CM, monthly dosing consisted of fremanezumab 675 mg (three 225 mg injections) at baseline followed by single 225 mg/1.5 mL injection at Weeks 4 and 8. The study protocols were reviewed and approved by the appropriate institutional review board for each site. The clinical trials were conducted in accordance with Good Clinical Practice and the Declaration of Helsinki guidelines. Participants provided written informed consent prior to study initiation.

### Post hoc analyses

The proportion of participants with migraine who responded to fremanezumab treatment were examined via post hoc analyses. Based on a ≥ 25% reduction in mean migraine days among the HALO study populations (EM: 8.9–9.3 days; CM: 16.0–16.2 days), responders were defined as participants with a reduction of ≥ 2 monthly migraine days in those with EM or ≥ 4 monthly migraine days in those with CM during the 12-week treatment period.

Among EM responders and CM responders, the efficacy of fremanezumab was measured by the reduction from baseline in the MAMD and days with acute headache medication use. Additional analyses included the proportion of participants achieving ≥ 50% and ≥ 75% reductions from baseline in the MAMD during the 12-week treatment period.

The impacts of fremanezumab on disability and HRQoL among responders were also evaluated. Headache-related disability in participants with EM was measured using the Migraine Disability Assessment (MIDAS) questionnaire, which assesses headache-related disability based on lost days of activity over the preceding 3 months [12]. The 6-item Headache Impact Test (HIT-6) was used to measure headache-related disability in participants with CM [13]. The 14-item Migraine-Specific Quality of Life (MSQoL) questionnaire was used to measure the emotional effects of migraine (emotional function domain) as well as the degree to which migraine limits (role function–restrictive domain) and prevents (role function–preventive domain) the performance of normal activities; this questionnaire was used to evaluate the effect of fremanezumab treatment on individuals’ HRQoL in both EM and CM [14].

### Statistical analysis

Due to the exploratory nature, the efficacy endpoints were analyzed using descriptive statistics for the responder subgroup compared to the overall study population. Descriptive statistics for continuous variables included count (n), mean, standard deviation (SD), standard error, median, minimum, and maximum. Descriptive statistics for categorical variables included patient counts and percentages.

## Results

**Fig. 1:**
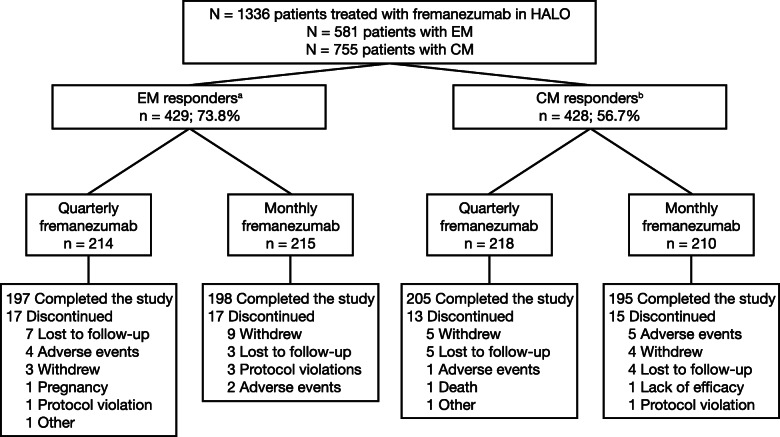
Patient disposition. CM, chronic migraine; EM, episodic migraine; MAMD, monthly average migraine days. ^a^For participants with EM, response was defined as a reduction of ≥2 MAMD. ^b^For participants with CM, response was defined as a reduction of ≥4 MAMD

The HALO trials included 1336 participants treated with fremanezumab (581 subjects with EM and 755 subjects with CM). Of these, 857 participants were responders according to the defined thresholds: 429 (73.8%) participants with EM had a reduction of ≥ 2 MAMD, and 428 (56.7%) participants with CM had a reduction of ≥ 4 MAMD (Fig. 1). Of these 857 participants, 395 (92.1%) participants with EM and 400 (93.5%) participants with CM completed the study. Demographics and baseline clinical characteristics were generally similar among participants with EM and CM and across treatment groups (Table 1). The baseline MAMD for EM responders was 9.3 (SD: 2.5) days for subjects receiving fremanezumab quarterly and 9.1 (2.6) days for those receiving fremanezumab monthly. The baseline MAMD was comparable for EM nonresponders at 9.1 (2.9) and 8.4 (2.6) days for quarterly and monthly dosing, respectively. CM responders receiving fremanezumab quarterly and monthly had 16.3 (4.6) and 16.0 (4.7) MAMD, respectively, at baseline. Similarly, among CM nonresponders, the baseline average number of migraine days was 16.1 (5.3) and 16.0 (5.8) days per month for fremanezumab quarterly and monthly, respectively.

**Table 1:** Demographics and baseline characteristics for participants who responded to treatment

Characteristic	EM responders^**a**^		CM responders^**b**^	
	Quarterly fremanezumab (***n*** = 214)	Monthly fremanezumab (***n*** = 215)	Quarterly fremanezumab (***n*** = 218)	Monthly fremanezumab (***n*** = 210)
Age, mean ± SD, y	41.4 ± 11.7	43.1 ± 12.2	41.9 ± 12.7	41.3 ± 11.9
Body mass index, mean ± SD, kg/cm^2^	26.9 ± 5.2	26.3 ± 5.4	26.7 ± 5.3	26.5 ± 5.0
Male sex, n (%)	29 (14)	34 (16)	22 (10)	24 (11)
**Race, n (%)**				
White	171 (80)	178 (83)	171 (78)	171 (81)
Black/African American	21 (10)	17 (8)	20 (9)	16 (8)
Asian	20 (9)	18 (8)	22 (10)	21 (10)
Other	2 (< 1)	2 (< 1)	5 (2)	2 (< 1)
**Disease history**				
Years since initial migraine diagnosis, mean ± SD	19.9 ± 11.9	20.5 ± 12.8	20.0 ± 12.6	19.8 ± 11.2
Current preventive medication use, n (%)	40 (19)	44 (20)	41 (19)	45 (21)
Current use of acute headache medication, n (%)	209 (98)	212 (99)	210 (96)	199 (95)
**Disease characteristics**				
Monthly migraine days,^c^ mean ± SD	9.3 ± 2.5	9.1 ± 2.6	16.3 ± 4.6	16.0 ± 4.7
Headache days of any severity,^d^ mean ± SD	8.5 ± 3.2	8.1 ± 3.2	15.5 ± 5.7	15.5 ± 5.5
Monthly days of acute headache medication use, mean ± SD	7.9 ± 3.6	7.9 ± 3.3	13.0 ± 6.6	13.3 ± 6.9
**MIDAS score**	*n* = 201	*n* = 205		
Mean ± SD, points	39.2 ± 32.1	37.0 ± 33.4	N/A	N/A
**HIT-6 score**			*n* = 204	*n* = 202
Mean ± SD, points	N/A	N/A	64.3 ± 5.0	64.9 ± 4.5
**MSQoL domain score, mean ± SD, points**	*n* = 211	*n* = 212	*n* = 215	*n* = 208
EF	64.9 ± 23.2	65.5 ± 24.4	57.3 ± 26.4	58.3 ± 25.6
RFP	71.0 ± 17.9	71.7 ± 17.6	66.2 ± 21.1	67.3 ± 21.3
RFR	56.5 ± 16.3	57.3 ± 16.0	47.7 ± 18.6	49.0 ± 18.9

*CM* Chronic migraine, *EF* Emotional function, *EM* Episodic migraine, *HIT-6* The 6-item Headache Impact Test, *MIDAS* Migraine Disability Assessment, *MSQoL* Migraine-Specific Quality of Life, *N/A* Not applicable, *RFP* Role function–preventive, *RFR* Role function–restrictive, *SD* Standard deviation, *MAMD* Monthly average migraine days

^a^Responders are defined as participants with EM who had a reduction of ≥2 MAMD

^b^Responders are defined as participants with CM who had a reduction of ≥4 MAMD

^c^A migraine day was defined as a calendar day in which headache pain lasted ≥4 consecutive hours and met criteria for migraine (with or without aura) or probable migraine (subtype in which only 1 migraine criterion is absent), or a day in which acute migraine–specific medication (triptans or ergots) was used to treat a headache of any duration

^d^A headache day was defined as a calendar day in which headache pain lasted at least 4 consecutive hours and had a peak severity of at least a moderate level, or a day in which acute migraine–specific medication (triptans or ergots) was used to treat a headache of any severity or duration

**Fig. 2:**
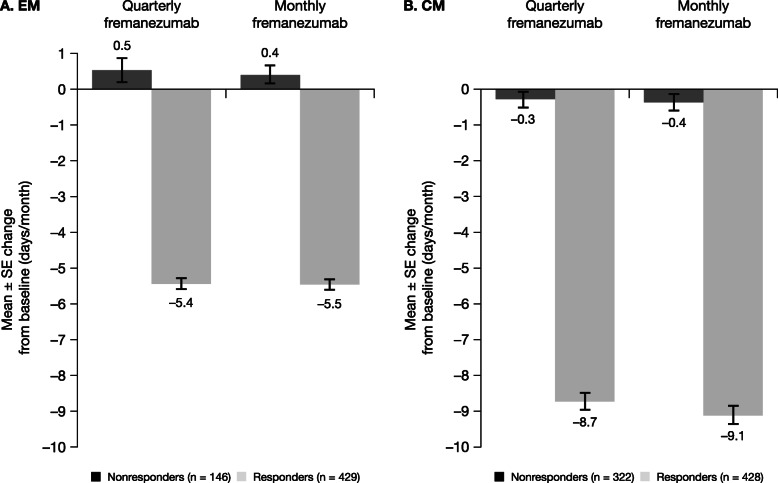
Mean change from baseline in the MAMD among participants with (**a**) EM^a^ and (**b**) CM^b^. CM, chronic migraine; EM, episodic migraine; MAMD, monthly average migraine days; SE, standard error. ^a^For participants with EM, response was defined as a reduction of ≥2 MAMD. ^b^For participants with CM, response was defined as a reduction of ≥4 MAMD

Over 12 weeks of treatment, the MAMD in EM responders decreased by 5.4 days with fremanezumab quarterly and by 5.5 days with fremanezumab monthly (58.5% and 60.2% reductions, respectively; Fig. 2). These reductions were greater than those observed in the overall EM population (quarterly: − 3.4 days, 37.0% reduction; monthly: − 3.7 days, 41.6% reduction). Among CM responders, the MAMD decreased by 8.7 days with fremanezumab quarterly and by 9.1 days with fremanezumab monthly during the study period (53.7% and 57.0% reductions, respectively). These reductions were greater than those in the overall CM population (quarterly: − 4.9 days, 30.2% reduction; monthly: − 5.0 days, 31.3% reduction). Smaller changes from baseline were observed in nonresponders: EM (quarterly: 0.53 days, 5.9% increase; monthly: 0.40 days, 4.8% increase) and CM (quarterly: − 0.28 days, 1.7% reduction; monthly: − 0.37 days, 2.3% reduction).

**Fig. 3:**
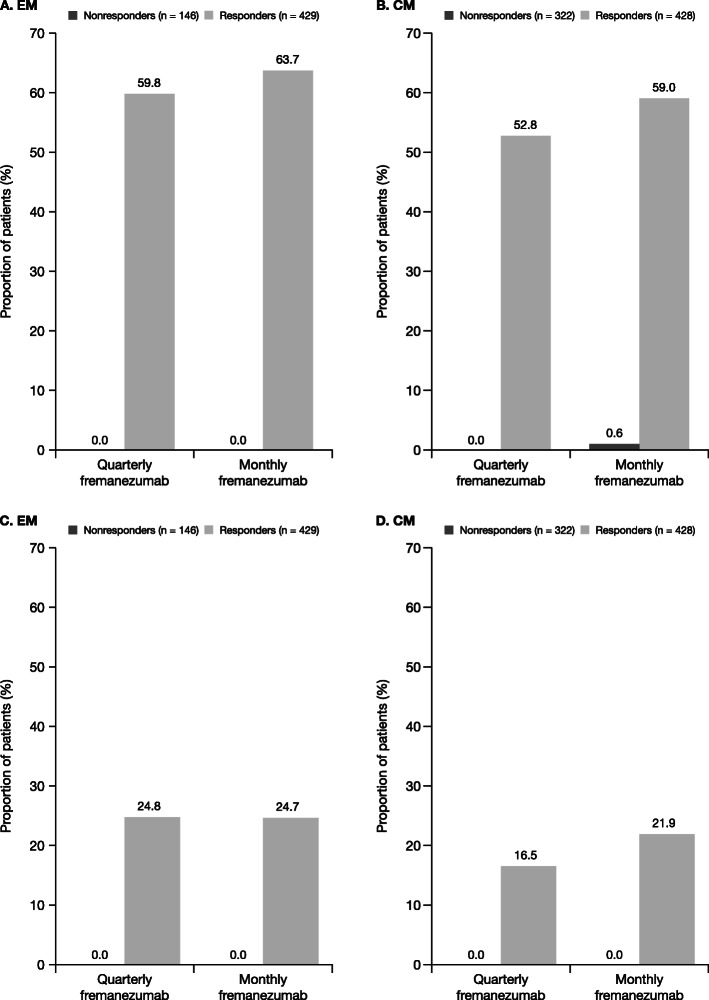
Proportion of participants^a,b^ with ≥ 50% (**a**, **b**) or ≥ 75% (**c**, **d**) reduction in MAMD. CM, chronic migraine; EM, episodic migraine; MAMD, monthly average migraine days; SE, standard error. ^a^For participants with EM, response was defined as a reduction of ≥ 2 MAMD. ^b^For participants with CM, response was defined as a reduction of ≥ 4 MAMD

The proportion of subjects with ≥ 50% reduction in the MAMD among responders receiving fremanezumab quarterly and monthly was 59.8% and 63.7%, respectively, for EM and 52.8% and 59.0%, respectively, for CM, compared with < 1% for all EM and CM nonresponders (Fig. 3a and b). Response rates (≥ 50% reduction) were higher in responders than in the overall population (EM: quarterly, 44.4%; monthly, 47.7%; CM: quarterly, 37.6%; monthly, 40.8%). The proportion of subjects receiving fremanezumab quarterly and monthly, who achieved a ≥ 75% reduction in the MAMD was 24.8% and 24.7%, respectively, in EM responders and 16.5% and 21.9%, respectively, in CM responders (Fig. 3c and d); these rates were higher than those observed in the overall study populations (18.4% and 18.5%, respectively, for EM and 9.6% and 12.3%, respectively, for CM).

**Fig. 4:**
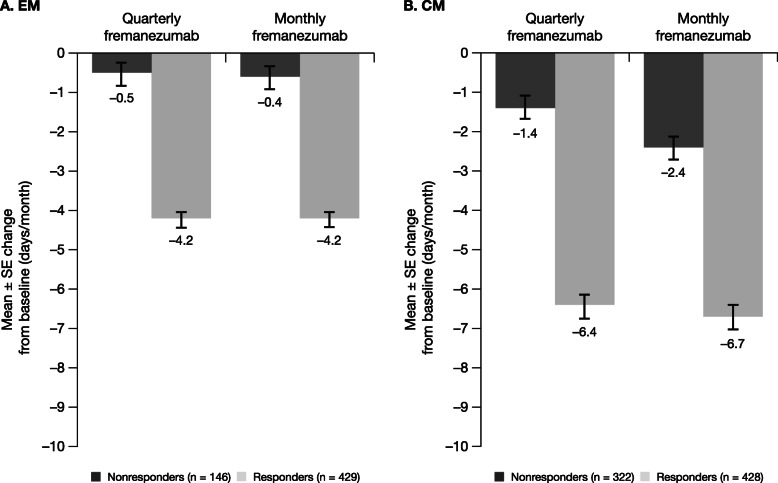
Mean change from baseline in monthly average number of days with any acute headache medication use. ^**a, b**^ CM, chronic migraine; EM, episodic migraine; MAMD, monthly average migraine days. ^a^For participants with EM, response was defined as a reduction of ≥ 2 MAMD. ^b^For participants with CM, response was defined as a reduction of ≥ 4 MAMD

Both fremanezumab dose regimens were associated with greater reductions in the monthly average number of days of acute headache medication use in EM responders (quarterly: − 4.2 days; monthly: − 4.2 days) compared with EM nonresponders (quarterly: − 0.5 days; monthly: − 0.6 days; Fig. 4a). A similar pattern was observed for CM responders (quarterly: − 6.4 days; monthly: − 6.7 days) compared with CM nonresponders (quarterly: − 1.4 days; monthly: − 2.4 days; Fig. 4b). Greater reductions in the monthly average number of days of acute headache medication use were also observed in EM and CM responders than in the overall populations (EM: quarterly: − 2.9 days; monthly: − 3.0 days; CM: quarterly: − 3.7 days; monthly: − 4.2 days).

**Fig. 5:**
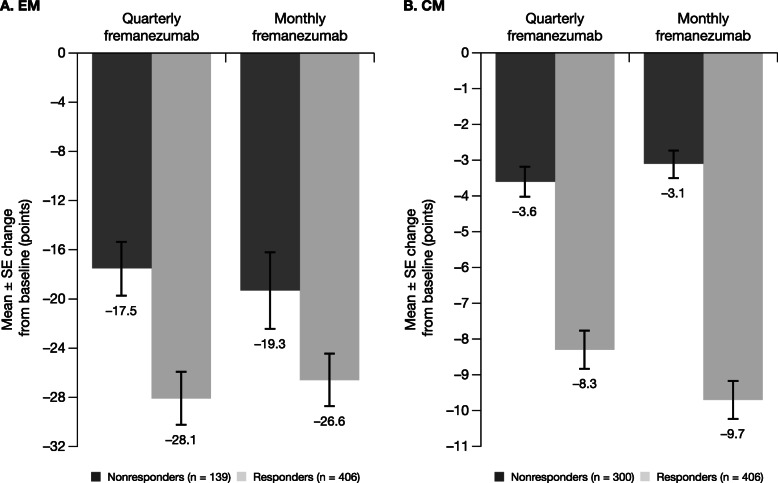
Mean change from baseline in headache-related disability, as measured by (**a**) MIDAS^a^ and (**b**) HIT-6^b^. CM, chronic migraine; EM, episodic migraine; HIT-6, 6-item Headache Impact Test; MAMD, monthly average migraine days; MIDAS, Migraine Disability Assessment; SE, standard error. ^a^For participants with EM, response was defined as a reduction of ≥ 2 MAMD. ^b^For participants with CM, response was defined as a reduction of ≥ 4 MAMD

**Fig. 6:**
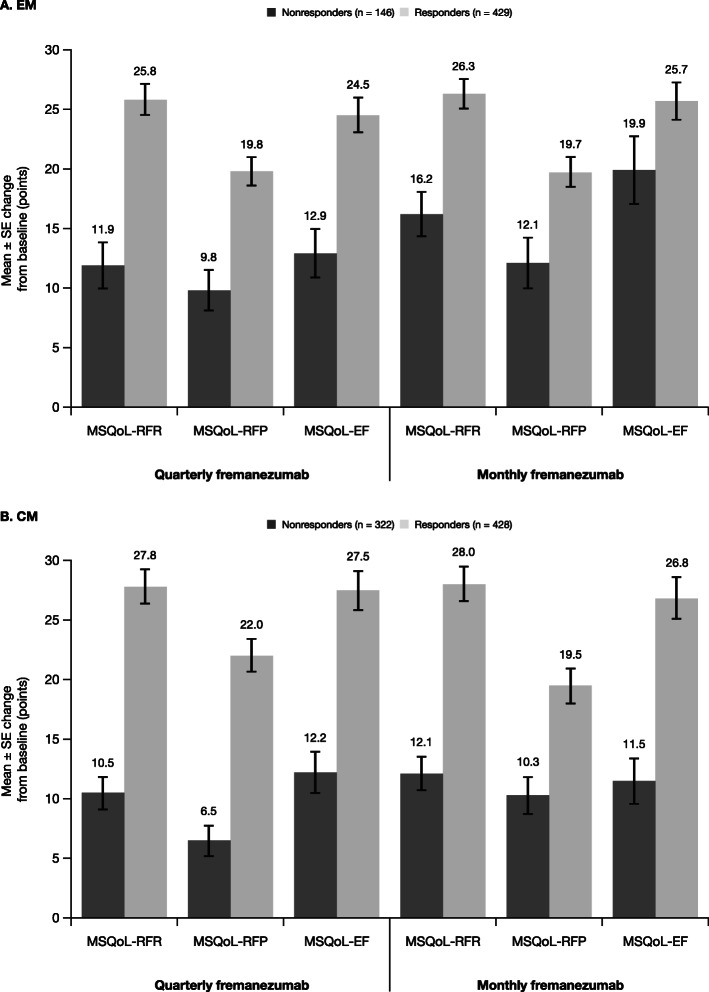
Mean change from baseline in MSQoL in participants with (**a**) EM^a^ and (**b**) CM^b^. CM, chronic migraine; EM, episodic migraine; MAMD, monthly average migraine days; MSQoL, Migraine-Specific Quality of Life; MSQoL-EF, MSQoL emotional function; MSQoL-RFR, MSQoL role function–restrictive; MSQoL-RFP, MSQoL role function–preventive; SE, standard error. ^a^For participants with EM, response was defined as a reduction of ≥ 2 MAMD. ^b^For participants with CM, response was defined as a reduction of ≥ 4 MAMD

Treatment with both fremanezumab dose regimens led to substantial reductions in headache-related disability scores in EM and CM responders. Different instruments for measuring disability were used in subjects with EM and in those with CM: in EM subjects, the MIDAS was used, while in CM subjects, the HIT-6 was used. Among EM responders, MIDAS score reductions from baseline were − 28.1 points with fremanezumab quarterly and − 26.6 points with fremanezumab monthly (Fig. 5a), which were greater than those observed in the overall EM population (quarterly: − 23.0 points; monthly: − 24.6 points). CM responders had improvements in headache-related disability, with HIT-6 score reductions from baseline of − 8.3 points and − 9.7 points with fremanezumab quarterly and monthly dosing, respectively. These improvements in HIT-6 score were greater than those observed in the overall CM population (quarterly: − 6.4 points; monthly: − 6.8 points). Reductions in both MIDAS and HIT-6 scores were of lower magnitude among nonresponders with EM (quarterly: − 17.5 points; monthly: − 19.3 points) and CM (quarterly: − 3.6 points; monthly: − 3.1 points). Responders with EM and CM demonstrated large improvements from baseline in all 3 MSQoL domains; nonresponders reported much smaller improvements across MSQoL domains (Fig. 6). Improvements in MSQoL scores were greater in EM and CM responders than in the overall population.

## Discussion

These post hoc analyses of the pivotal HALO clinical trials demonstrate that fremanezumab is associated with substantial improvements in migraine frequency, headache-related disability, and HRQoL in a significant proportion of participants with EM and CM who respond to treatment. A considerable proportion of participants responded to fremanezumab treatment when using study-specific thresholds of a reduction of ≥2 monthly migraine days for participants with EM (quarterly: 73.5%; monthly: 74.1%) and ≥ 4 MAMD for participants with CM (quarterly: 58.0%; monthly: 55.4%). A reduction of ≥ 4 MAMD aligns with a response rate of ≥ 30%, which is considered clinically meaningful for participants with CM [4]. Although the reduction of ≥ 2 MAMD (≥ 25%) for participants with EM is lower than the generally accepted threshold for a clinically meaningful response (≥ 50%), this threshold was intended to capture the broader range of participants with EM included in the study, some of whom could have as few as 4 MAMD [10]. Using a higher threshold to define responders would have further increased the magnitude of effect in these outcomes, but this lower threshold allows for a distinction between effect in those with little to no response from those with a clinically meaningful improvement. The large variance between efficacy outcomes in subgroups divided at this 25% threshold suggests this is a reasonable cut point to differentiate responders from nonresponders.

This post hoc analysis demonstrates that people who respond to fremanezumab show greater improvements in all outcomes than those observed in the overall trial populations. Markedly greater reductions in the MAMD were observed in EM responders (59%–60% reduction) and CM responders (54%–57%) than in the total study populations (37%–42% and 30%–32%, respectively). Response rates (≥ 50% and ≥ 75% reduction in the MAMD) were also higher in responders than in the overall populations, and EM and CM responders had greater reductions in the monthly average number of days of acute headache medication use. Improvements in headache-related disability scores (MIDAS and HIT-6) and HRQoL (MSQoL) were also greater in EM and CM responders than in the overall population.

Overuse of acute headache medications must be considered in the treatment and long-term management of people with migraine. Among people with CM, acute medication overuse is associated with increased risk of developing medication overuse headache (MOH), which is also associated with poorer prognosis, additional comorbidities, greater disability, and further reduced HRQoL among people with CM [15–19]. According to ICHD-3 criteria, MOH develops as a consequence of regular overuse of acute or symptomatic headache medication for more than 3 months on ≥ 10 days per month for ergot derivatives, triptans, opioids, or combination analgesics or on ≥ 15 days per month for nonopioid analgesics, acetaminophen, or nonsteroidal anti-inflammatory drugs [20]. In our analysis, the average number of days per month in which acute headache medication was used in EM responders was 7.9 days at baseline and 3.7 days after 12 weeks of fremanezumab treatment (52.9%–53.7% reduction) and in CM responders was 13.0 to 13.3 days at baseline and 6.6 days after 12 weeks of fremanezumab treatment (49.3%–50.4% reduction). This suggests that people with EM or CM who respond to fremanezumab use less acute headache medication, which may lead to resolution of MOH or reduce the risk of developing MOH.

Positive patient-centered outcomes, such as headache-related disability and HRQoL, show significant improvement in subjects treated with fremanezumab. EM and CM responders had greater reductions in headache-related disability than EM and CM nonresponders. The improvements in HIT-6 scores observed in CM responders (reductions of 8.3 and 9.7 points with fremanezumab quarterly and monthly dosing, respectively) exceeded the minimal clinically important difference for HIT-6 score in participants with chronic daily headache (2.3 points) [21]. Furthermore, compared to the nonresponder groups, the responder groups on quarterly and monthly dosing achieved greater reductions that exceeded the minimal clinically important difference in HIT-6 scores. This observation highlights the meaningful clinical benefit achieved between responder and nonresponder CM participants, as defined in this study. For CM responders, participants in the quarterly fremanezumab group experienced a shift in HIT-6 scores from grade 4 (severe impact; HIT-6 score of 60–78) to grade 3 (substantial impact; HIT-6 score of 56–59), while those in the monthly fremanezumab group experienced a shift from grade 4 to grade 2 (moderate impact; HIT-6 score of 50–55) after 12 weeks of therapy. EM responders had substantial improvements in MIDAS scores (72% reduction), with shifts from baseline scores that indicated severe disability (MIDAS score ≥ 21) to end-of-treatment scores suggesting only mild disability (MIDAS score ~ 10) after fremanezumab treatment. According to the American Headache Society consensus statement, evidence of treatment benefit with anti-CGRP monoclonal antibodies includes a clinically meaningful improvement in a validated migraine-specific patient-reported outcome measure, such as a 30% reduction in MIDAS score for those with baseline scores above 20 or a reduction of at least 5 points on the HIT-6, [22] both of which were achieved by fremanezumab responders in the current analysis. EM and CM responders also had greater improvements in all 3 domains of the MSQoL compared with nonresponders, which suggests that people who respond to treatment with fremanezumab have reduced disability and increased HRQoL.

The results from this study may help clinicians to manage patient expectations when using fremanezumab for the preventive treatment of migraine. People who are responsive to fremanezumab have large reductions in migraine days and headache days of at least moderate severity, reductions in acute headache medication use, and improvements in disability as well as overall HRQoL. Persistence, defined as the ability of a patient to continue on treatment over an extended period of time, is poor with older migraine preventive medications [23, 24]. Lack of efficacy, long titration periods, and delayed onset of efficacy contribute to poor persistence for many migraine medications [25]. In contrast, responders to fremanezumab had an early and robust reduction in migraine frequency, which may encourage participants to continue with treatment. Adherence may also be improved with the choice of quarterly or monthly dosing when compared with dosing once or multiple times a day, which is known to produce poor adherence in migraine [26].

There are several limitations to consider when interpreting the results from these analyses. This study involved post hoc analyses of data from 2 separate 12-week, placebo-controlled, phase 3 trials and, thus, may not be adequately powered or designed to characterize treatment response, and the post hoc determination of subgroup definitions should be interpreted with caution. However, since assessments were performed a priori, the evaluation of efficacy outcomes in responders may still provide directional guidance for clinicians seeking to advise participants on expected treatment effect if they respond to fremanezumab. Furthermore, the duration of the 12-week HALO clinical trials is not sufficient for understanding the longer-term or sustained benefits of fremanezumab treatment. As such, additional studies are necessary to evaluate the maintenance of response over longer treatment periods.

## Conclusions

In conclusion, participants with EM or CM who responded to fremanezumab over the 12-week treatment period demonstrated clinically meaningful response rates, reductions in the frequency of migraine days, fewer days with acute headache medication use, and improvements in headache-related disability and HRQoL, and the magnitude of these benefits was much greater than that observed in the overall trial population. The results from this study will help to inform clinicians’ decision making and provide guidance for patients regarding treatment expectations.

## Data Availability

Qualified researchers may request access to patient-level data and related study documents, including the study protocol and the statistical analysis plan. Requests will be reviewed for scientific merit, product approval status, and conflicts of interest. Patient-level data will be de-identified, and study documents will be redacted to protect the privacy of trial participants and to protect commercially confidential information. Please email USMedInfo@tevapharm.com to make your request.

## Abbreviations

CGRP: Calcitonin gene-related peptide
CM: Chronic migraine
EF: Emotional function
EM: Episodic migraine
HIT-6: 6-item Headache Impact Test
HRQoL: Health-related quality of life
ICHD-3 beta: International Classification of Headache Disorders, 3rd edition beta
MAMD: Monthly average migraine days
MIDAS: Migraine Disability Assessment
MOH: Medication overuse headache
MSQoL: Migraine-Specific Quality of Life
RFP: Role function–preventive
RFR: Role function–restrictive
SD: Standard deviation
SE: Standard error

## References

[1] Tepper SJ (2018). History and review of anti-calcitonin gene-related peptide (CGRP) therapies: from translational research to treatment. Headache..

[2] Hoy SM (2018). Fremanezumab: first global approval. Drugs..

[3] Tassorelli C, Diener HC, Dodick DW, Silberstein SD, Lipton RB, Ashina M (2018). Guidelines of the international headache society for controlled trials of preventive treatment of chronic migraine in adults. Cephalalgia..

[4] Silberstein S, Tfelt-Hansen P, Dodick DW, Limmroth V, Lipton RB, Pascual J (2008). Guidelines for controlled trials of prophylactic treatment of chronic migraine in adults. Cephalalgia..

[5] Silberstein SD (2015). Preventive migraine treatment. Continuum (Minneap Minn).

[6] AJOVY® (fremanezumab-vfrm) injection [packet insert]. North Wales: Teva Pharmaceuticals USA, Inc.; 2019

[7] AJOVY® (fremanezumab) [Summary of Product Characteristics]. Ulm: Teva Pharmaceuticals GmbH; 2019

[8] Bigal ME, Edvinsson L, Rapoport AM, Lipton RB, Spierings EL, Diener HC (2015). Safety, tolerability, and efficacy of TEV-48125 for preventive treatment of chronic migraine: a multicentre, randomised, double-blind, placebo-controlled, phase 2b study. Lancet Neurol.

[9] Bigal ME, Dodick DW, Rapoport AM, Silberstein SD, Ma Y, Yang R (2015). Safety, tolerability, and efficacy of TEV-48125 for preventive treatment of high-frequency episodic migraine: a multicentre, randomised, double-blind, placebo-controlled, phase 2b study. Lancet Neurol.

[10] Dodick DW, Silberstein SD, Bigal ME, Yeung PP, Goadsby PJ, Blankenbiller T (2018). Effect of fremanezumab compared with placebo for prevention of episodic migraine: a randomized clinical trial. JAMA..

[11] Silberstein SD, Dodick DW, Bigal ME, Yeung PP, Goadsby PJ, Blankenbiller T (2017). Fremanezumab for the preventive treatment of chronic migraine. N Engl J Med.

[12] Lipton RB, Stewart WF, Sawyer J, Edmeads JG (2001) Clinical utility of an instrument assessing migraine disability: the Migraine Disability Assessment (MIDAS) questionnaire. Headache. 41:854–861 11703471

[13] Rendas-Baum R, Yang M, Varon SF, Bloudek LM, DeGryse RE, Kosinski M (2014) Validation of the Headache Impact Test (HIT-6) in patients with chronic migraine. Health Qual Life Outcomes 12:117 10.1186/s12955-014-0117-0 PMC4243819 25080874

[14] Bagley CL, Rendas-Baum R, Maglinte GA, Yang M, Varon SF, Lee J (2012). Validating migraine-specific quality of life questionnaire v2.1 in episodic and chronic migraine. Headache..

[15] Kristoffersen ES, Lundqvist C (2014). Medication-overuse headache: epidemiology, diagnosis and treatment. Ther Adv Drug Saf.

[16] Lanteri-Minet M, Duru G, Mudge M, Cottrell S (2011). Quality of life impairment, disability and economic burden associated with chronic daily headache, focusing on chronic migraine with or without medication overuse: a systematic review. Cephalalgia..

[17] Probyn K, Bowers H, Caldwell F, Mistry D, Underwood M, Matharu M (2017). Prognostic factors for chronic headache: a systematic review. Neurology..

[18] Vandenbussche N, Laterza D, Lisicki M, Lloyd J, Lupi C, Tischler H (2018). Medication-overuse headache: a widely recognized entity amidst ongoing debate. J Headache Pain.

[19] Da Silva AN, Lake AE (2014). Clinical aspects of medication overuse headaches. Headache..

[20] Headache Classification Committee of the International Headache Society (IHS) (2018) The International Classification of Headache Disorders, 3rd edition. Cephalalgia. 38:1–211 10.1177/0333102417738202 29368949

[21] Coeytaux RR, Kaufman JS, Chao R, Mann JD, Devellis RF (2006) Four methods of estimating the minimal important difference score were compared to establish a clinically significant change in Headache Impact Test. J Clin Epidemiol 59:374–380 10.1016/j.jclinepi.2005.05.010 16549259

[22] American Headache Society (2019) The American Headache Society position statement on integrating new migraine treatments into clinical practice. Headache. 59:1–18 10.1111/head.13456 30536394

[23] Hepp Z, Dodick DW, Varon SF, Chia J, Matthew N, Gillard P (2017). Persistence and switching patterns of oral migraine prophylactic medications among patients with chronic migraine: a retrospective claims analysis. Cephalalgia..

[24] Hepp Z, Bloudek LM, Varon SF (2014). Systematic review of migraine prophylaxis adherence and persistence. J Manag Care Pharm.

[25] Blumenfeld AM, Bloudek LM, Becker WJ, Buse DC, Varon SF, Maglinte GA et al (2013) Patterns of use and reasons for discontinuation of prophylactic medications for episodic migraine and chronic migraine: results from the second International Burden of Migraine Study (IBMS-II). Headache. 53:644–655 10.1111/head.12055 23458496

[26] Cowan R, Cohen JM, Rosenman E, Iyer R (2019). Physician and patient preferences for dosing options in migraine prevention. J Headache Pain.

